# Alterations of Electrophysiological Properties and Ion Channel Expression in Prefrontal Cortex of a Mouse Model of Schizophrenia

**DOI:** 10.3389/fncel.2019.00554

**Published:** 2019-12-17

**Authors:** Zhen Mi, Jun Yang, Quansheng He, Xiaowen Zhang, Yujie Xiao, Yousheng Shu

**Affiliations:** ^1^State Key Laboratory of Cognitive Neuroscience and Learning, Beijing Normal University, Beijing, China; ^2^IDG/McGovern Institute for Brain Research, Beijing Normal University, Beijing, China

**Keywords:** schizophrenia, ion channel, neuronal excitability, action potential, prefrontal cortex, SK channel, Nav channel

## Abstract

Maternal immune activation (MIA) and juvenile social isolation (SI) are two most prevalent and widely accepted environmental insults that could increase the propensity of psychiatric illnesses. Using a two-hit mouse model, we examined the impact of the combination of these two factors on animal behaviors, neuronal excitability and expressions of voltage-gated sodium (Nav) and small conductance calcium-activated potassium (SK) channels in the prefrontal cortex (PFC). We found that MIA-SI induced a number of schizophrenia-related behavioral deficits. Patch clamp recordings revealed alterations in electrophysiological properties of PFC layer-5 pyramidal cells, including hyperpolarized resting membrane potential (RMP), increased input resistance and enhanced medium after-hyperpolarization (mAHP). MIA-SI also increased the ratio of the maximal slope of somatodendritic potential to the peak slope of action potential upstroke, indicating a change in perisomatic Nav availability. Consistently, MIA-SI significantly increased the expression level of Nav1.2 and SK3 channels that contribute to the somatodendritic potential and the mAHP, respectively. Together, these changes may alter neuronal signaling in the PFC and behavioral states, representing a molecular imprint of environmental insults associated with neuropsychiatric illnesses.

## Introduction

Schizophrenia is a chronic and disabling psychiatric disorder affecting approximately 1% of the population worldwide (Owen et al., 2016). It is assumed that a single adverse event is rather unlikely to cause schizophrenia. Instead, the two-hit hypothesis suggests that a prenatal genetic or environmental first-hit can prime an individual for an adverse event later (second-hit) in life that ultimately provides substantial triggers for the full clinical syndrome to manifest (Bayer et al., 1999; Maynard et al., 2001; Feigenson et al., 2014). There is ample evidence that maternal immune activation (MIA) can act as a disease primer to increase the risk of neuropsychiatric disorders in offspring (Feigenson et al., 2014; Davis et al., 2016; Estes and McAllister, 2016), while social deprivation during adolescence has been thought to work as a second-hit leading to behavioral and biochemical changes that feature some aspects of schizophrenia (Jones et al., 2011; Makinodan et al., 2012; Davis et al., 2016; Yamamuro et al., 2017). As two most prevalent and widely accepted environmental insults, MIA and social isolation (SI) can commonly occur together in life. However, research investigating the impact of MIA-SI combination is very limited.

Functions of prefrontal cortex (PFC) primarily rely on electrochemical signals generated by its constituent neurons. Disruptions of these signals have been shown in schizophrenia. Enhanced PFC neuronal excitability was found in Disrupted in schizophrenia 1 (*DISC1*) mutant mouse model of schizophrenia (Crabtree et al., 2017). Two-week SI after weaning results in reductions in intrinsic excitability of a subpopulation of pyramidal cells (PCs) in mouse PFC (Yamamuro et al., 2017). Both NMDA receptor antagonist MK801 and serotonergic agonist DOI are psychotogenic drugs, which cause transient states of psychosis. Interestingly, MK801 increased PFC population activity, whereas DOI decreased population activity (Wood et al., 2012). It remains unclear whether the MIA-SI two-hit would cause any changes in the intrinsic excitability of PCs in PFC.

Given the significance of ion channels in the regulation of neuronal excitability and synaptic plasticity, it’s not surprising that altered ion channel expression and function would cause severe disruptions in brain functions, thus contributing to schizophrenia etiology (Smolin et al., 2012). Voltage-gated sodium channels (Nav) are particularly critical for the initiation and propagation of action potentials (APs). Cortical PCs express two Nav subtypes, i.e., low-threshold Nav1.6 and high-threshold Nav1.2 channels, which preferentially accumulate at distal and proximal regions of the axon initial segment (AIS), respectively, (Tian et al., 2014). Distal Nav1.6 triggers AP initiation, whereas proximal Nav1.2 promotes AP backpropagation to the soma (Hu et al., 2009). Polymorphisms of *SCN2A*, the gene encoding Nav1.2, are associated with the occurrence of schizophrenia (Dickinson et al., 2014; Carroll et al., 2016). Mutation of *SCN8A*, which encodes Nav1.6, is also involved in the susceptibility of suicidal behavior among psychiatric disorder patients (Wang et al., 2010). Moreover, Nav channel blockers can be used as mood stabilizers, antidepressants and antipsychotics (Imbrici et al., 2013). Therefore, Nav channels may be a valuable target for effective treatments. However, alterations in Nav channels in animal model of schizophrenia have not been characterized yet.

Previous studies also revealed important roles of potassium channels in schizophrenia (Shepard et al., 2007; Vukadinovic and Rosenzweig, 2012; Georgiev et al., 2014; Yanagi et al., 2014). For example, the small conductance calcium-activated potassium (SK) channels, which mediate the medium after-hyperpolarization (mAHP) (Villalobos et al., 2004), may play a role in the etiology of schizophrenia and related cognitive disorders (Faber and Sah, 2007). The gene *KCNN3* encoding SK3 locates at 1q21, a chromosome closely related to schizophrenia (Gargus, 2006). Polymorphism of *KCNN3*, which reduces SK3 channel function, is associated with enhanced cognitive performance in schizophrenia patients (Grube et al., 2011). In contrast, overexpression of SK3 channel induces hippocampal shrinkage and cognitive deficits (Martin et al., 2017). Inhibition of SK2 channels also improves learning and memory (Lam et al., 2013). It remains unknown whether SK channels change in animal models of schizophrenia.

In this study, we used peripheral administration of immunostimulant Polyinosinic:polycytidylic acid (poly I:C) in pregnant dam combined with juvenile isolation rearing to model schizophrenia in mice. We examined the consequences of MIA and early life SI on animal behaviors and electrophysiological properties of layer 5 (L5) PCs in prelimbic (PL) and infralimbic (IL) regions of PFC, which associates with multiple emotional, cognitive, and mnemonic functions. We also assessed the impacts of MIA-SI on the expression of Nav and SK channels.

## Materials and Methods

### Animals

**FIGURE 1:**
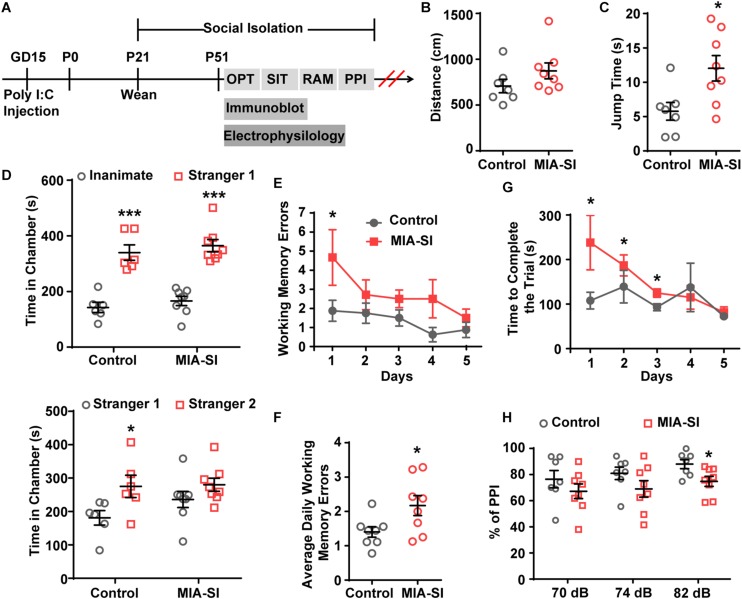
MIA-SI animals develop schizophrenia-like behaviors, including deficits in jumping behavior, social novelty preference, working memory and sensorimotor gating function. **(A)** Overview of the experimental design. OFT, open field test; SIT, social interaction test; RAM, radial arm maze; PPI, prepulse inhibition. **(B)** Total distance traveled was not changed by MIA-SI in OFT. **(C)** Increased jumping time was observed in MIA-SI animals in OFT. **(D)** Top, time spent in the non-social chamber (inanimate) versus the social chamber (stranger 1) during the sociability session in SIT, indicating MIA-SI didn’t affect sociability. Bottom, time spent in the chamber with familiar mouse (stranger 1) versus that with novel mouse (stranger 2) during social novelty preference session in SIT, suggesting MIA-SI caused deficit in social novelty preference. **(E)** Working memory errors committed across five test days in RAM test. **(F)** Average daily working memory errors over five test days was increased in MIA-SI animals. **(G)** Time to complete the trial across five test days in RAM test. **(H)** PPI deficits following poly I:C challenge and isolation rearing. ^∗^*p* < 0.05; ^∗∗∗^*p* < 0.001. Control, *n* = 7; MIA-SI, *n* = 8.

Protocols of all animal experiments were approved by the Animal Advisory Committee at the State Key Laboratory of Cognitive Neuroscience and Learning, Beijing Normal University. The experiments were conducted in accordance with the guidelines set out by the Animal Advisory Committee. We obtained pregnant C57BL/6 mice from Beijing Vital River Laboratory Animal Technology Co., Ltd., (Beijing, China). Intraperitoneal injection of 5 mg/kg poly I:C (Tocris, Bristol, United Kingdom) or saline were carried out at gestational day 15 (GD15). Their offspring were weaned at postnatal day 21 (P21) and only male littermates were used for the following experiments. Offspring of saline-treated animals were housed in groups of 3–5 animals per cage, whereas the offspring of poly I:C-treated animals were reared individually for a month (Figure 1A). In consequence, we obtained two animal groups, control group, i.e., saline treated-social animals, and MIA-SI group, i.e., poly I:C treated-isolated animals. All animals were kept in a controlled environment (22 ± 2°C and 12-h light/darkness cycle). Water and standard pellet chow was available *ad libitum*. Separate cohorts of mice were used for behavioral tests, immunoblots and electrophysiological recordings to avoid the influences of behavioral paradigms on animals.

### Animal Behavior

#### Open-Field Test

Spontaneous locomotor activity and general behavior were evaluated using an automated open-field apparatus (Med Associates Inc., Fairfax, VT, United States). The apparatus consists of a 27.31 × 27.31 × 20.32 cm^3^ transparent seamless chamber with an open roof, a sound attenuating cubicle with venting and lighting, and a computer. 16-beam infrared arrays located on both the *X* and *Y* axes for positional tracking and *Z* axis for rearing detection. The animals were placed individually in the center of the open-field arena and recorded for 5 min. General locomotor activity was assessed by measuring total distance traveled, as well as total duration of jumps. Total distance traveled is the total Euclidean distance of all ambulatory episodes in centimeters. Jump is when *Z*-axis infrared beam breaks were detected while no *X* or *Y*-axis beam breaks were detected. The jump time is the total duration of the jumping behavior.

#### Sociability and Social Novelty Preference Test

The apparatus used in this test consists of clear acrylic walls, an opaque gray bottom, and two clear partitions which divide the apparatus into three chambers of same size. Each partitions had one door in the center which could be lifted up to form a passage between chambers. One outer chamber was designated as social chamber randomly, and the other outer chamber was designated novelty chamber. Each outer chamber had a wire cage to entrap one stranger mouse. A video camera was fixed above the apparatus to record the activity of animals and was connected to a monitor and behavioral tracking system (ANY-maze, Stoelting Co., Wood Dale, IL, United States). The sociability and social novelty preference test protocols were adapted from previous study with slight modifications (Moy et al., 2004). The protocol contains three phases that occur in sequence: habituation, sociability, and social novelty preference. The day before the test, all experimental mice and stranger mice were allowed to acclimate to the test room and apparatus. On the test day, the experimental mouse was firstly placed in the center chamber and allowed to freely explore the three chambers for 5 min. Immediately after the habituation phase, the stranger mice 1 was placed in the wire cage in the social chamber, while the wire cage in the other outer chamber still stayed empty. Then the test mouse was allowed to freely access to two wire cages for another 10 min. After this sociability session, stranger mice 2 was introduced into the novelty chamber, and the test mouse was allowed to explore for another 10 min. The sociability and social novelty preference of the test mouse were determined by measuring the time spent in social chamber and novelty chamber during all three phases.

#### Radial Arm Maze for Working Memory Test

We used an automated eight arm radial maze (Med Associates Inc., United States) consisting of eight runways with dual IR sensors for each arm, pellet receptacles with head entry detector at the end of each runway, and eight automatic guillotine doors separating the hub area from each of the eight arms. Before experiments, mice were weighed and fasted for 24 h, and 2–3 g pellet chow were given to each animals daily on following test days. Animals were then habituated to the apparatus for three consecutive days. During habituation, mice were placed in the hub and allowed to explore the maze for 10 min per day. Baits were scattered on the arms on the first habituation day, and then gradually reduced and located toward the end of each runway. Following habituation, the animals were tested one session per day for five consecutive days. For each trial, all eight arms were baited with one food reward in food receptacle at the end of the arms. Experimental subjects were first placed in the hub area for 2 min with all guillotine doors closed. Then all eight doors raised, and animals could explore the maze to collect baits. Re-entering a visited arm was defined as one working memory error. Each session ended when all eight arms had been entered or 10 min passed since the door opened. The variables used for the analyzing the performance of the mice are the number of errors in each trial (day), the time taken to complete the trial on each day, and the average daily number of errors throughout 5 days.

#### Prepulse Inhibition

Prepulse inhibition (PPI) was measured using an acoustic startle reflex system (Med Associates Inc., United States) as described previously (Hadar et al., 2018). Briefly, to measure startle reflex, we set the background noise level to 66 dB. The 100 ms startling pulse (110 dB) was preceded by a 20-ms-long non-startling prepulse (70, 74 or 82 dB in a random order). PPI for a given prepulse intensity was calculated as percent inhibition of the startle response using the following formula:

$$\text{PPI}=100-\frac{\mathrm{average}\,\mathrm{startle}\,\mathrm{response}\,\mathrm{for}\,\mathrm{PPI}\,\mathrm{trials}}{\mathrm{average}\,\mathrm{startle}\,\mathrm{response}\,\mathrm{for}\,\mathrm{startle}-\mathrm{only}\,\mathrm{trials}}\times 100$$

### Immunoblot

Protein samples were isolated from the PFC tissue blocks and separated on 8% SDS polyacrylamide gels. The primary antibodies are as follows: mouse anti-Nav1.2, clone K69/3, 1:200 (Neuromab, Davis, CA, United States); rabbit anti-Nav1.6, 1:200; rabbit anti-SK1, #APC-039, 1:200; rabbit anti-SK2, #APC-028, 1:200; rabbit anti-SK3 N-term, #APC-025, 1:200 (Alomone Labs, Jerusalem, Israel); mouse anti-α-tubulin, clone DM1A, 1:1000 (Sigma-Aldrich, St. Louis, MO, United States). We used 3 and 4 animals for control and MIA-SI groups, respectively. And experiments were repeated for 3–4 times. The integrated density of samples on blots were analyzed using ImageJ (National Institutes of Health, United States). For each blot, we normalized the integrated density of each sample to β-tubulin and then normalized this ratio to the mean ratio of samples in control groups. Then we averaged the normalized density of each sample, and performed statistical analysis (Student’s *t*-test).

### Slice Preparation

Coronal slices of PFC were obtained from experimental mice as previously described (Hu et al., 2009). Animals were anesthetized with sodium pentobarbital (50 mg/kg) and perfused with ice-cold sucrose-based artificial cerebrospinal fluid (ACSF) followed by decapitation. The brains were then dissected out and slices with a thickness of 300 μm were cut in sucrose-based ACSF using a vibratome (VT-1200S, Leica, IL, United States). Then brain slices were immediately transferred to an incubation chamber filled with normal ACSF and maintained at 34.5°C for 30 min in water bath. For recording, individual slices were transferred to a recording chamber perfused with normal ACSF at 34.5–35.5°C for whole-cell recording. An infrared-differential interference contrast (IR-DIC) microscope (BX-51WI, Olympus) was used for visualizing individual cells in the slice. The normal ACSF contained (in mM) 126 NaCl, 2.5 KCl, 2 MgSO_4_, 2 CaCl_2_, 26 NaHCO_3_, 1.25 NaH_2_PO_4_ and 25 dextrose (315 mOsm, pH 7.4), and was bubbled continuously with carbogen.

### Electrophysiological Recordings

We performed whole-cell patch-clamp recordings from L5 PCs in PL and IL regions of PFC as described previously (Hu et al., 2009). Patch pipettes had an impedance of 4–7 MΩ with the normal internal solution contained (in mM) 140 K-Gluconate, 3 KCl, 2 MgCl_2_, 0.2 EGTA, 10 HEPES, 2 Na_2_ATP (pH 7.2–7.25, 285–295 mOsm). Recordings were performed using a Multiclamp 700B amplifier (Molecular Devices, San Jose, CA, United States). We used Micro1401-3 together with Spike2 software (version 8) (Cambridge Electronic Design Limited, Cambridgeshire, United Kingdom) for data acquisition. Voltage signals were filtered at 10 kHz and sampled at 50 kHz. The liquid junction potential (15.2 mV) was not corrected for the data shown in the text and figures. All electrophysiological recordings were performed and analyzed blindly.

Resting membrane potential (RMP) was defined as membrane potential measured when no current injected. Rheobase was defined as the threshold current that elicits a single AP. The AP threshold was defined as the voltage at which the time derivative of membrane potential (dV/dt) reached 20 V/s. AP half width (HW) is the duration of the AP at half amplitude from AP threshold. Amplitude of mAHP is measured as difference between AP threshold and the trough in a time window of 100 ms after individual APs. The sag ratio was measured from the hyperpolarization induced by negative current pulse injections (amplitude: −100 pA). It is the ratio of voltage difference between the peak and the steady-state of hyperpolarization to the peak amplitude. We considered the PCs with sag ratio >5% as those with prominent H-current. The mAHP, threshold, peak amplitude, half width and maximum depolarization and repolarization slope were measured from the single APs elicited by rheobase current, while Slope_SD_ ratio was measured from the first APs in 7-AP trains. The parameters were analyzed using MATLAB R2017b (MathWorks Inc., Natick, MA, United States).

### Statistical Analysis

All data in figures and tables are presented as mean ± SEM. In our study, we considered the combination of MIA and SI as a single factor. Therefore, we performed the two-tailed unpaired Student’s *t*-test to compare two normally distributed sample groups and Mann–Whitney U test to compare two non-normally distributed groups. A value of *p* < 0.05 was considered significant. Statistical analysis was carried out using OriginPro-9 (OriginLab Corp., Northampton, MA, United States) and SigmaPlot 14.0 (Systat Software Inc., San Jose, CA, United States).

## Results

### MIA-SI Causes Deficits in Locomotor Activity, Social Novelty Preference, Working Memory and Sensorimotor Gating Function

We carried out four behavioral paradigms to examine whether the animals develop schizophrenia-like behaviors. Animals was assessed on behavioral tasks in the following sequence: open-field tests, sociability and social novelty preference test, radial arm maze, and PPI (Figure 1A). Tasks were separated by 3–7 rest days to ensure minimum interference between tasks.

Hyperactivity has been demonstrated in many different putative animal models of schizophrenia (Powell et al., 2009). We initially examined the locomotor activity in these animals. MIA-SI didn’t influence the total distance traveled by these animals (Figure 1B). However, we observed substantially longer jump time (*p* < 0.05, Student’s *t*-test) (Figure 1C) in MIA-SI animals. Jumping behavior has been examined as one of the basic locomotive behaviors in the open field test and homecage monitoring (Choleris et al., 2001; Huang et al., 2007; van Dam et al., 2013; Adamah-Biassi et al., 2014). An increase in jumping behavior has been considered as an indicator of hyperactivity and increased exploratory behavior (Hashimoto et al., 2001; Shintani et al., 2006). Therefore, the results indicate that MIA-SI animals were more hyperactive.

Sociability and social novelty test was often used for assessing the social behavior in animal models of schizophrenia (Powell and Miyakawa, 2006). Within the sociability session, both groups exhibited preference for the stranger mice over the inanimate chamber (*p* < 0.001) (Figure 1D, top). In the social novelty preference phase, results varied between groups: control animals appeared to interact more with the novel conspecific stranger (*p* < 0.05), while the MIA-SI group showed no significant preference for the novel stranger versus the familiar conspecific (*p* = 0.177) (Figure 1D, bottom). These results indicate that MIA-SI disrupted the animal’s preference for social novelty but not animal’s sociability.

We next examined the working memory of these animals using a classic eight-arm radial maze paradigm (Powell and Miyakawa, 2006). Working memory errors committed on day 1 was increased in MIA-SI group (*p* < 0.05, Figure 1E). MIA-SI also significantly increased the average daily working memory errors over five consecutive days of test (*p* < 0.05, Figure 1F). The time taken to complete the trial was significantly longer in MIA-SI animals from day 1 to day 3 (*p* < 0.05, Figure 1G). These results suggest that environmental insults jeopardize the working memory of animals.

Prepulse Inhibition deficit is another common symptom of schizophrenia (Powell et al., 2009). We found MIA-SI had no effects on startle reflex at 110 dB (data not shown). PPI at prepulse stimulus intensities of 70 and 74 dB was not affected by MIA-SI (Figure 1H). However, there was a significant difference in PPI between the two groups with prepulse stimulus at 82 dB (*p* < 0.05, Figure 1H), indicating disrupted sensorimotor gating in this two-hit animal model of schizophrenia. Altogether, our results suggest that MIA-SI two-hit animals exhibit abnormalities in schizophrenia relevant behaviors.

### MIA-SI Hyperpolarizes RMP, and Increases Input Resistance and mAHP

**FIGURE 2:**
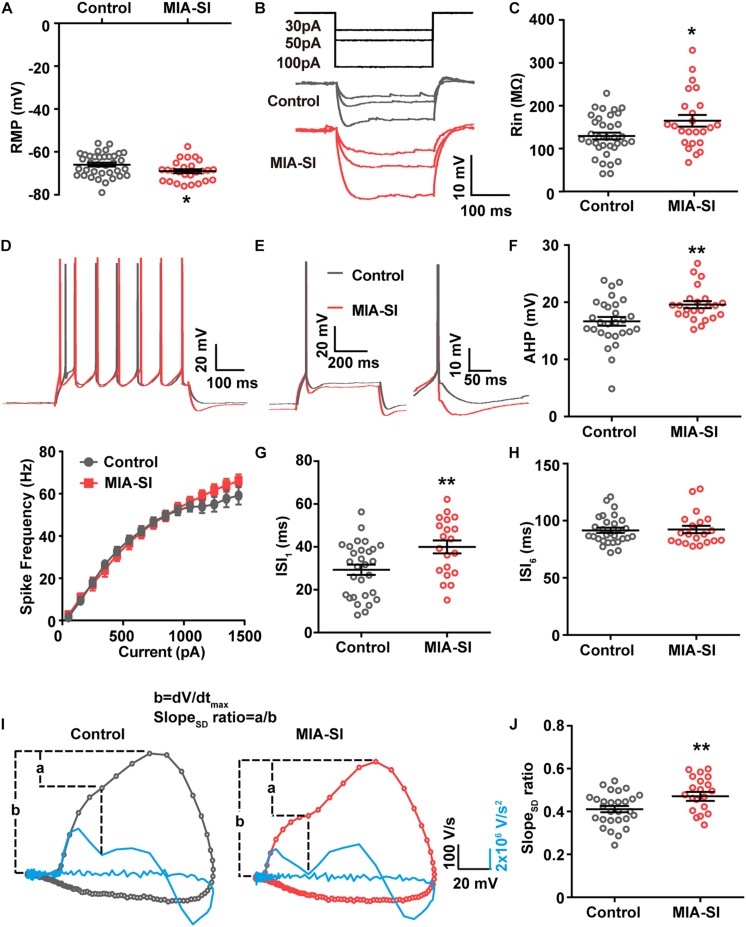
MIA-SI induces alterations in electrophysiological properties in PCs, including hyperpolarized RMP, and increased input resistance, mAHP and AP phase ratio. **(A)** Group data showed that MIA-SI caused hyperpolarization of RMP. **(B)** Example voltage traces of PCs in response to hyperpolarizing current pulses. **(C)** Group data of input resistance (Rin) showed MIA-SI increased Rin in L5 PCs. **(D)** Top, representative 7-AP traces of PCs elicited by current injection in control and MIA-SI groups; Bottom, Input-output (I-F curve) relationships of neurons from two experimental groups were not significantly different. **(E)** Representative full traces (left) and enlarged traces (right) showing changes in mAHP of single AP evoked by threshold current. **(F)** Group analysis showed MIA-SI increased mAHP amplitude. The first inter-spike interval (ISI_1_) **(G)** but not the last inter-spike interval (ISI_6_) **(H)** of 7-AP trains was increased by MIA-SI. **(I)** Representative phase plots and the second derivatives of the voltage waveforms (shown in blue) for control (left) and MIA-SI (right) PCs. The contribution of SD potential slope to the peak slope is calculated by the ratio of a/b. **(J)** Group data of Slope_SD_ ratio. ^∗^*p* < 0.05; ^∗∗^*p* < 0.01.

Juvenile SI alters intrinsic properties of deep layer PCs in PFC (Yamamuro et al., 2017). We next performed whole-cell recordings from L5 PCs in PL and IL regions of PFC. Analysis showed that MIA-SI caused a significant hyperpolarizing change in the RMP (*p* < 0.05, Figure 2A). Cells of the two-hit group also showed a greater input resistance (Rin) in contrast to the control group (*p* < 0.05, Figures 2B,C). To examine the effects of MIA-SI on neuronal firing, we injected a series of incremental depolarizing current pulses from 50 pA to 1,500 pA into the recorded cells (example traces shown in Figure 2D, top panel) and found no significant difference in the firing frequency between the two groups (Figure 2D, bottom panel). We then closely examined the properties of single APs evoked by threshold current (i.e., rheobase). The threshold current and the latency to spike was not affected by MIA-SI (Table 1). The peak amplitude of mAHP was significantly increased in the two-hit group (*p* < 0.01, Figures 2E,F). Other parameters of the single APs evoked by rheobase were not altered by MIA-SI (Table 1). To test the spike accommodation, we analyzed the first and sixth inters-pike interval (ISI_1_ and ISI_6_) in 7-AP trains (Figure 2G). The currents evoking 7 AP were not different between control (median, 230.0 pA) and MIA-SI (median, 250.0 pA) neurons (*p* = 0.433). The mAHP of AP_1_ to AP_6_ were all increased in MIA-SI group. However, the increase in AHP of AP_1_ was larger than other APs (Supplementary Figure S1). There is a substantial increase in ISI_1_ (*p* < 0.01, Student’s *t*-test, Figure 2H) but not in ISI_6_ (*p* = 0.9, Mann–Whitney test, Figure 2I). Such change leaded to a significant decrease in AP accommodation ratio, i.e., (ISI_6_-ISI_1_)/ISI_6_. These results indicate MIA-SI affected spike accommodation of these PCs.

**TABLE 1:** Electrophysiological parameters of L5 PCs in control and MIA-SI animals.

	**Control**	**MIA-SI**	***p-*value**
RMP (mV)	−65.9 ± 0.8(*n* = 36)	−69.0 ± 0.9(*n* = 26)	0.024
Rin (MΩ)	130 ± 8(*n* = 35)	165 ± 14(*n* = 24)	0.019
Rheobase (pA)	110 ± 15(*n* = 36)	133 ± 18(*n* = 26)	0.91
Spike latency (ms)	180 ± 17(*n* = 24)	176 ± 19(*n* = 20)	0.88
Threshold (mV)	−33.5 ± 1.0(*n* = 28)	−34.4 ± 1.1(*n* = 23)	0.50
Peak amplitude (mV)	80.3 ± 2.5(*n* = 28)	78.7 ± 2.8(*n* = 23)	0.77
Half width (ms)	0.88 ± 0.05(*n* = 28)	0.85 ± 0.05(*n* = 23)	0.64
mAHP (mV)	16.7 ± 0.7(*n* = 28	19.6 ± 0.8(*n* = 23)	0.009
dV/dt_max_ (mV/ms)	391 ± 27(*n* = 28)	391 ± 29(*n* = 23)	0.99
dV/dt_min_ (mV/ms)	−90.5 ± 5.0(*n* = 28)	−88.5 ± 5.5(*n* = 23)	0.78

*dV/dtmax, maximum depolarization slope; dV/dtmin, maximum repolarization slope; *n*, number of neurons.*

The rising phase of somatic APs contains two components as revealed by the phase plots (Figure 2I), the AIS potential and the somatodendritic (SD) potential (Hu et al., 2009). The SD potential is generated mainly by perisomatic Nav channels (i.e., Nav1.2). Second derivative (d^2^V/dt^2^) of the AP waveforms showed a trough in the AP rising phase, reflecting the breakpoint between the AIS potential and SD potential (Kole, 2011). We defined the contribution of SD potential to the AP rising phase by the ratio of the maximal slope of SD potential (a) to the peak slope of AP upstroke (b) (Figure 2J). MIA-SI didn’t cause significant alterations in either a or b-a. However, analysis showed that MIA-SI significantly increased the ratio of a to b, suggesting that the perisomatic Nav may be changed (*p* < 0.01, Figure 2J).

Previous studies showed that 2-week isolation immediately after weaning reduces the intrinsic excitability only in a subtype of L5 PCs with prominent H-current in PFC (Yamamuro et al., 2017). Therefore, we also analyzed the aforementioned electrophysiological parameters of the neurons showing prominent H-current. Control and MIA-SI groups were not different in sag ratio (Supplementary Figure S2). 54.3% PCs in control group (*n* = 19 out of 35) and 54.2% PCs (*n* = 13 out of 24) in MIA-SI group exhibit prominent H-current. We obtained similar results in electrophysiological properties from these cells (Supplementary Figure S3 and Supplementary Table S1). In conclusion, our results revealed that MIA-SI two-hit animal model displayed deficits in neuronal electrophysiological properties.

### MIA-SI Increases the Expression Levels of Nav1.2 and SK3 Channels

**FIGURE 3:**
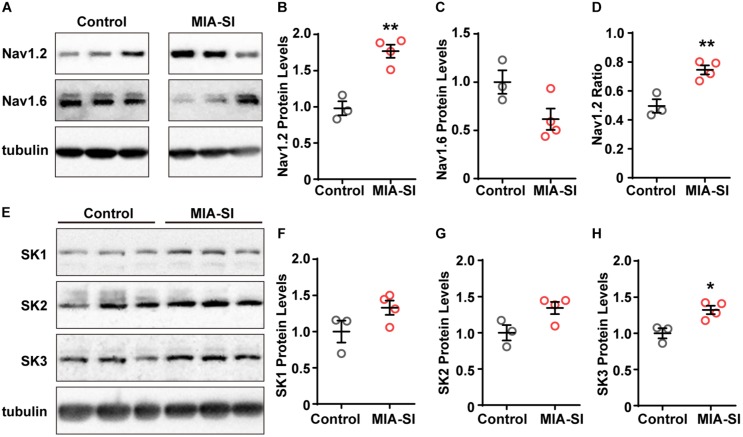
MIA-SI increases the expression levels of Nav1.2 and SK3 channels. **(A)** Representative immunoblots of Nav1.2, Nav1.6 and tubulin, run on a same gel. Each band represents samples from one animal. **(B,C)** Average Nav1.2 and Nav1.6 protein levels in PFC in two experimental groups. **(D)** Averaged ratio of Nav1.2 to the sum of Nav1.2 and Nav1.6 was increased by MIA-SI. **(E)** Representative immunoblots of SK1, SK2, SK3, and tubulin run on a same gel. **(F–H)** Average SK1, SK2, and SK3 protein levels in PFC of experimental animals. For each protein, blots were cropped to retain the important bands at the same size of the same membrane. ^∗^*p* < 0.05; ^∗∗^*p* < 0.01.

The channel subtype Nav1.6 triggers AP initiation, whereas Nav1.2 mainly contributes to AP backpropagation to the soma (Hu et al., 2009) and form the SD potential. Therefore, we hypothesized that the alterations in the amount or function of Nav1.2 may cause changes in the SD potential slope ratio. To test this hypothesis, we firstly examined the protein expression of Nav1.2 and Nav1.6. Quantification demonstrated that Nav1.2 protein levels were significantly increased by MIA-SI (*p* < 0.01, Figures 3A,B). In contrast, MIA-SI tended to decrease Nav1.6 at an insignificant level (Figures 3A,C). We analyzed the ratio of Nav1.2 to the sum of Nav1.2 and Nav1.6, and found Nav1.2 percentages were increased by MIA-SI (*p* < 0.01, Figure 3D), indicating that the proportion of Nav1.2 and Nav1.6 was influenced by MIA and SI.

The increased mAHP amplitude in MIA-SI group could be attributable to changes in the expression level of SK channels (Villalobos et al., 2004). To test this assumption, we also examined the protein levels of SK1, SK2, and SK3 channels. We found that SK1 and SK2 protein levels exhibited an increasing tendency in MIA-SI animals but without statistical significance (Figures 3E–G). In contrast, MIA-SI significantly increased SK3 (*p* < 0.05) expression levels in PFC (Figure 3H). These results indicate that MIA can cause abnormal ion channel expression.

## Discussion

In this work, we investigated behavioral and neurophysiological alterations in animals challenged with two environmental insults, MIA and SI. We found that combining the MIA with SI exacerbates a number of schizophrenia relevant behaviors, including deficits in locomotor activity, social novelty preference, working memory and sensorimotor gating function. Moreover, MIA and SI together also altered neurophysiological properties of L5 PCs in PFC, including RMP, input resistance, mAHP and spike accommodation. The ratio of the maximal slope of SD potential to the peak slope of AP upstroke is also increased by MIA-SI, indicating a change in perisomatic sodium channels. Indeed, immunoblot experiments revealed an increase in Nav1.2 and SK3 channels, consistent with the increases in the somatodendritic potential slope ratio and the amplitude of mAHP, respectively. These results indicate that MIA and SI mediate adverse effects on animal behaviors and neuronal electrophysiological properties.

Immunostimulant challenges and stressors provoke both common and distinct effects, and mediate synergistic effects on some animal behaviors and neurochemistry (Monte et al., 2017). Previous MIA-stress two-hit studies uses different animals (e.g., age, sex, species), dose and timing of poly I:C exposure, as well as behavioral paradigms, thus leading to some discrepant results of behavioral tests (Gandhi et al., 2007; Lukasz et al., 2013; Feigenson et al., 2014; Monte et al., 2017). For example, a study combining the postnatal poly I:C challenge with SI found that MIA-SI induced PPI deficit at 72, 76, 80 and 84 dB (Lukasz et al., 2013), but we only found MIA-SI-induced PPI deficits at 82 dB. This difference may in part results from different dose and timing (P38–46 vs. E15) of poly I:C treatment.

We observed an increase in jumping behavior in MIA-SI mice. We assumed this change was an indicator of hyperactivity and increased exploratory behavior based on previous studies (Hashimoto et al., 2001; Shintani et al., 2006). However, jumping behavior is also a prominent sign of physical dependence during morphine withdrawal in mice (David and Cazala, 2000), which could be related to anxiety, considering that anxiety is one of the common psychological symptoms of morphine withdrawal. However, in mice lacking pituitary adenylate cyclase-acting polypeptide (PACAP), augmented jumping behavior in OFT and lower anxiety level shown in elevated plus maze and novel-object test were discovered simultaneously (Hashimoto et al., 2001). Therefore, we could not conclude that increased jumping behavior was due to anxiety. In the current study, we mainly focused on the core symptoms of schizophrenia. However, early adversities, including both MIA and SI, can induce diverse emotional and behavioral deficits, including anxiety-related behaviors (Babri et al., 2014; Canetta et al., 2016; Rincel et al., 2019). Therefore, it’s very likely that one would see changes in MIA-SI animals if their anxiety-like behaviors were tested.

A previous study showed that 2-week SI immediately after weaning reduced the intrinsic excitability of L5 PCs in PFC, reflected by an increase in spike threshold and a decrease in firing frequency (Yamamuro et al., 2017). However, our experiments revealed no changes in spike threshold and the input-output curve (i.e., F-I curve). In our research, we found that the input resistance was substantially increased, which should lead to enhanced neuronal excitability. However, we also observed more hyperpolarized RMP and greater mAHP in the two-hit group, which may decrease the intrinsic excitability. These alterations may counteract with each other and consequently result in no change in firing frequency. For example, we found a prolonged ISI accompanied with increased mAHP, which could offset the increase in the input resistance. This phenomenon could be the mechanism underlying the adaptation to stresses.

An increase in input resistance could be caused by less channels per unit membrane area, which may result from a reduction in total channel amount or an increase in neuron size. We compared the sag ratio of these cells and found no difference, suggesting that HCN channels were not responsible for the changes in input resistance. Considering the major contribution of resting channels (e.g., resting potassium, chloride and sodium channels) to the membrane resistance, we speculate a change in their expression level or channel activity. However, this possibility remains to be examined in future studies.

Nav1.2 plays a pivotal part in mediating AP backpropagation, which is proposed to regulate synaptic plasticity, release of retrograde neurotransmitters and trophic factors, and spike-timing-dependent plasticity. It’s likely that an increase in Nav1.2 levels impacts upon these functions, thus disturbing PFC neural circuits and finally causing schizophrenia. Furthermore, compared to Nav1.2, Nav1.6 channels activate with more hyperpolarized membrane potentials and have a higher propensity to generate a non-inactivating persistent sodium current, which contributes to setting membrane potential in a subthreshold range (Astman et al., 2006). Indeed, conditional knockout of Nav1.6 leads to a strong reduction in persistent sodium current (Chen et al., 2018). In our experiments, we found a decrease in Nav1.6 in the MIA-SI group, providing an explanation for the hyperpolarization of the RMP.

Our results show that Nav1.2 protein levels were significantly increased, while Nav1.6 protein levels tended to be declined by MIA-SI, and the proportion of Nav1.2 was increased in MIA-SI animals (meaning that Nav1.6 proportion was also decreased significantly). The Nav1.6 changes could be a compensatory adaptation subsequent to Nav1.2 elevations. The minimal changes in maximum depolarization slope, which is mainly contributed by Nav, could result from such compensation. Besides, Nav1.2 is the only Nav isoform at AIS and nodes of Ranvier during early development, and replaced by Nav1.6 in the distal AIS subsequently (Ben-Shalom et al., 2017). Therefore, MIA-SI could influence early developmental transition of Nav1.2 during the course of postnatal maturation.

A growing body of evidence implicates several calcium and potassium channels in the susceptibility of schizophrenia, such as *CACNA1C* (Cav1.2 α subunit), *KCNN3* (SK3), *KCNH3* (Ether-A-Go-Go), and *KCNJ3* (Kir3.1) (Smolin et al., 2012; Imbrici et al., 2013). Among those channels, SK3 channel plays major roles in determining the mAHP amplitude, and represents a potential drug target to improve cognitive functions in schizophrenia. Our data showed a striking increase in mAHP amplitude in the two-hit group. We postulate that the increased expression of SK3 channels could contribute to the change in mAHP. We detected SK channel expression using total PFC tissue samples, which consist of different layers and types of neuronal cells, while we examined the electrophysiological properties only in deep layer PCs in PFC. Neocortical PCs express high levels of SK1 and SK2, and a relative low level of SK3 channels (Pedarzani and Stocker, 2008). Inhibitory neurons, glia cells and cerebral vasculature also contain SK channel expression (Pedarzani and Stocker, 2008; Dolga and Culmsee, 2012). Therefore, the immunoblot results of SK channels could also reflect the changes in other types of cells. Cell-specific expression of SK3 requires a further detection in future studies.

Although the evidence concerning ion channel alterations in the pathophysiology of psychiatric disorders are emerging, knowledge about schizophrenia as channelopathy is still limited. Our data suggests that Nav and SK channels are altered by maternal immune stimulation, and may in part contribute to the changes in intrinsic neuronal excitability properties. However, whether these channels contribute to the changes in electrophysiological properties eventually give rise to schizophrenia-like behaviors remains to be further examined.

## Data Availability Statement

The datasets generated for this study are available on request to the corresponding author.

## Ethics Statement

The animal study was reviewed and approved by the Animal Advisory Committee at the State Key Laboratory of Cognitive Neuroscience and Learning, Beijing Normal University.

## Author Contributions

ZM designed the study, completed the animal behavioral experiment, the whole-cell patch-clamp recordings, and Western blot experiments, managed the literature searches and data analyses, and wrote the first draft of the manuscript. JY and YX completed part of the whole-cell patch-clamp recordings. QH undertook the statistical analysis. XZ conducted part of the animal behavioral experiment. YS oversaw all aspects of study design, protocol development, and data collecting, and edited the manuscript. All authors contributed to and have approved the final version of the manuscript.

## Conflict of Interest

The authors declare that the research was conducted in the absence of any commercial or financial relationships that could be construed as a potential conflict of interest.
