# Supplementary material for: Alterations of Electrophysiological Properties and Ion Channel Expression in Prefrontal Cortex of a Mouse Model of Schizophrenia
Source: Front Cell Neurosci. 2019 Dec 17;13:554. doi: 10.3389/fncel.2019.00554 (PMC6927988; doi:10.3389/fncel.2019.00554)
Supplement: Supplementary file 1 [file Data_Sheet_1.PDF]

Supplementary Materials

1. Supplementary Figures

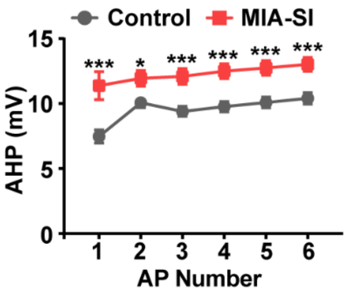

Figure S1. MIA-SI increased mAHP amplitude in 7-AP trains. Note that the increase in AHP of AP1 is largest. *, p<0.05; ***, p< 0.001.

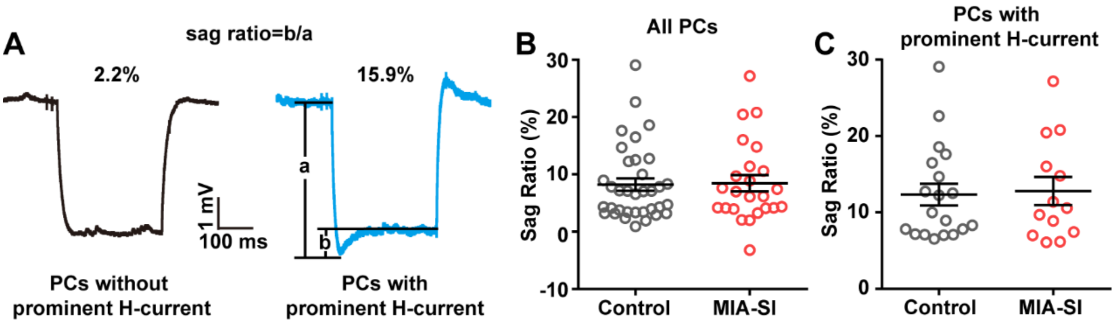

Figure S2. MIA-SI didn’t change sag ratio of PCs in PFC. (A) Representative traces of voltage responses to hyperpolarizing current pulse injections of -100 pA. Left, PC without prominent H-current. Right, PC with prominent H-current. (B) Group data of sag ratio in all PCs. (C) Group data of sag ratio in PCs with prominent H-current.

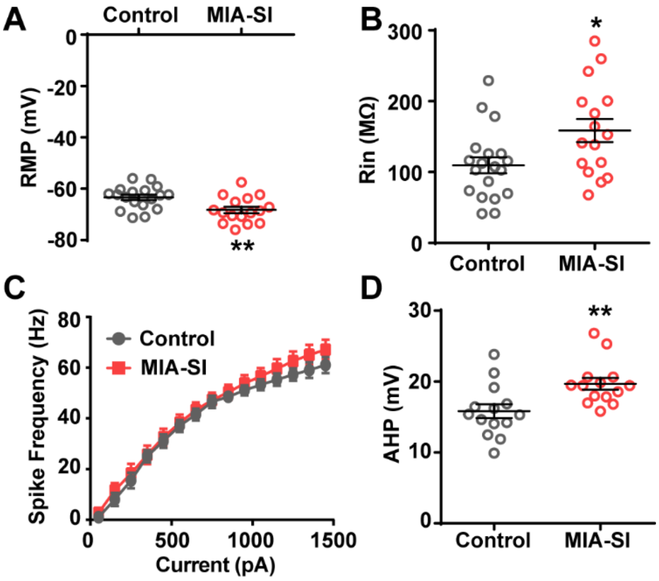

Figure S3. **MIA-SI had similar effects on electrophysiological properties in PCs with prominent H-current.** (A) Group data showed RMP hyperpolarization in MIA-SI animals. (B) Input resistance was increased by MIA-SI. (C) Input-output relationships of neurons. (D) Group data of the peak amplitude of mAHP was increased in MIA-SI PCs. *, p<0.05; **, p< 0.01.

2. Supplementary Tables

Table S1. Electrophysiological parameters of L5 PFC PCs with prominent H-current.

	Control	*MIA-SI*	*p value*
RMP (mV)	-63.4 ±1.0 (n=18)	-68.3 ±1.1 (n=16)	0.005
Rin (MΩ)	110 ±11 (n=19)	159 ±16 (n=16)	0.016
Rheobase (pA)	128 ±22 (n=18)	137 ±24 (n=16)	0.64
Threshold (mV)	-35.1 ±1.3 (n=14)	-34.2 ±1.3 (n=14)	0.54
Peak amplitude (mV)	84.2 ±3.3 (n=14)	82.3 ±3.3 (n=14)	0.60
Half width (ms)	0.80 ±0.06 (n=14)	0.87 ±0.06 (n=14)	0.45
mAHP (mV)	15.8 ±0.8 (n=14)	19.7 ±0.8 (n=14)	0.004
dV/dt_{max} *(mV/ms)*	436 ±36 (n=14)	419 ±36 (n=14)	0.75
dV/dt_{min} *(mV/ms)*	-101 ±7 (n=14)	-89.4 ±6.7 (n=14)	0.25
